# Healthy lifestyle behaviors and depressive symptoms: a national cross-sectional study of the older adults in China

**DOI:** 10.3389/fmed.2025.1548034

**Published:** 2025-02-25

**Authors:** Haojin Jiao, Shangjie Ge-Zhang, Jingqi Yang

**Affiliations:** ^1^School of Modern Post, Xi’an University of Posts and Telecommunications, Xi’an, China; ^2^College of Science, Northeast Forestry University, Harbin, China; ^3^Department of Cardiovascular Medicine, Jiangxi Provincial People’s Hospital, The First Affiliated Hospital of Nanchang Medical College, Nanchang, China

**Keywords:** China elderly population, depressive symptoms, healthy lifestyle behaviors, CHARLS, mental health

## Abstract

**Introduction:**

This investigation assesses the impact of healthy lifestyle behaviors on depressive symptoms among older adults in China, utilizing data from the 2020 China Health and Retirement Longitudinal Study (CHARLS).

**Methods:**

The analysis included 9,020 valid samples from individuals aged 60 and above. Sleep duration, social participation, and physical exercise were examined as independent variables. Depressive symptoms were measured using the CESD-10 scale, with relationships analyzed through an ordered logistic regression model.

**Results:**

The study reveals significant correlations between healthy lifestyle behaviors — specifically adequate sleep, regular physical exercise, and active social participation — and reduced prevalence of depressive symptoms in the elderly (*p* < 0.05).

**Discussion:**

These findings underscore the potential of healthy lifestyle interventions as key strategies in alleviating the mental health burden among China’s aging population. Integration of these results into public health policies is recommended to enhance the mental well-being of older adults.

## 1 Introduction

In recent years, population aging has become a significant global demographic trend, with China being no exception (1). As the elderly population grows, there is increasing societal concern regarding their physical and mental health (2). Among the various health issues, depression is a prevalent mental health risk factor, particularly for older adults. Symptoms of depression can severely impact quality of life and heighten the risk of adverse outcomes. Therefore, it is essential to investigate factors that may either alleviate or exacerbate these symptoms (3–5).

In recent social changes in China, rapid urbanization, economic transformation and changes in family structure have had a significant impact on the mental health of the elderly. The isolation measures during COVID-19 pandemic further aggravated these effects, which made the elderly face higher isolation risk and psychological pressure. Long-term isolation measures have brought serious challenges to the mental health of the elderly. The research shows that the prevalence of depression is particularly high among empty nesters, and compared with non-empty nesters, empty nesters are more likely to suffer from depression (6, 7). At the same time, the prevalence of depression among the elderly in China is high. Although the primary medical system is an ideal place to manage such problems, the current services are still difficult to meet the growing demand (8). In addition, the elderly’s self-awareness of their own depression is extremely low, which intensifies this challenge. Therefore, China’s public health policy urgently needs to integrate mental health services into the primary health care system, so as to improve the quality of life of the elderly and reduce the risk of depression caused by loneliness and social isolation (6, 9).

The epidemiological data of depression among the elderly in China show that the prevalence of depression among the elderly in the primary health care system is high, but the awareness of depression among this population is very limited (10). This emphasizes the importance of bringing mental health services into the primary health care system. In addition, studies have shown that factors related to depression include low education level, poor economic situation, family relationship problems, loneliness and chronic health problems (11, 12).

Although China is the first country to experience and control the COVID-19 epidemic, the demand for mental health services among its elderly population is increasing, and the existing public health strategies and infrastructure cannot effectively meet this challenge (8). Therefore, in view of the depression of the elderly, it is necessary to study and implement more effective public health intervention measures to improve the quality of life and mental health of this group (6, 9).

Healthy lifestyle behaviors have long been recognized as key contributors to promoting mental health. For older adults, specific behaviors such as regular physical activity, adequate sleep, and active social participation are commonly associated with improved mental well-being (13–15). However, the relationship between these behaviors and the incidence of depressive symptoms in the elderly, particularly in China, remains underexplored.

To address this research gap, the present study utilizes data from the 2020 China Health and Retirement Longitudinal Study (CHARLS 2020) (16), aiming to empirically examine the influence of various factors on the prevalence of depressive symptoms among individuals aged 60 and above. Understanding these relationships is crucial for developing intervention strategies to mitigate the mental health burden among China’s aging population.

## 2 Literature review

### 2.1 The global status and research of senile depression

The research shows that the depressive symptoms of the elderly are common all over the world. According to the report of the World Health Organization, about 7% of the global population aged 60 and above are affected by depression (17). Depression not only affects the quality of life of the elderly, but also may be related to cognitive decline, physical dysfunction and increased mortality. In China, due to the particularity of culture, social economy and family structure, the risk of depression faced by the elderly deserves more attention.

### 2.2 The relationship between healthy lifestyle and mental health

Current research generally suggests that a healthy lifestyle is closely linked to mental health. Examples include adequate sleep, active social engagement, and regular physical exercise. These behaviors have been shown to play a positive role in alleviating depressive symptoms. According to existing studies, both insufficient sleep and excessive sleep are significantly associated with an increased incidence of depressive symptoms. Additionally, some studies have indicated that maintaining regular social contact can reduce loneliness, thereby decreasing the occurrence of depressive symptoms (18–22).

### 2.3 The present situation of healthy lifestyle of the elderly in China

With the development of China’s economy and society, the lifestyle of the elderly is undergoing significant changes. Research indicates that although China continues to experience economic and social progress, the healthy lifestyle of its elderly population still requires improvement (23, 24). According to existing studies, many elderly individuals lack sufficient physical activity and social engagement, which may contribute to the exacerbation of mental health problems (25, 26).

### 2.4 The deficiency of existing research and the contribution of this study

Although the relationship between healthy lifestyle behaviors and depressive symptoms has been extensively studied, empirical research focused on the elderly population in China remains relatively limited, particularly in the area of in-depth analysis using large-scale national datasets. Therefore, this study comprehensively examines the influence of healthy lifestyle behaviors (such as sleep, social activities, and physical exercise) on depressive symptoms among the elderly in China, utilizing nationally representative data from CHARLS 2020, in conjunction with demographic factors. Compared to previous research, the contribution of this study lies in verifying and quantifying the specific impact of healthy lifestyle behaviors on depressive symptoms in the elderly using large-sample data, while also incorporating demographic characteristics, which are often overlooked in earlier studies. This study aims to provide empirical evidence for future research in this field (27–29).

### 2.5 Viewpoint hypothesis

**Figure 1:**
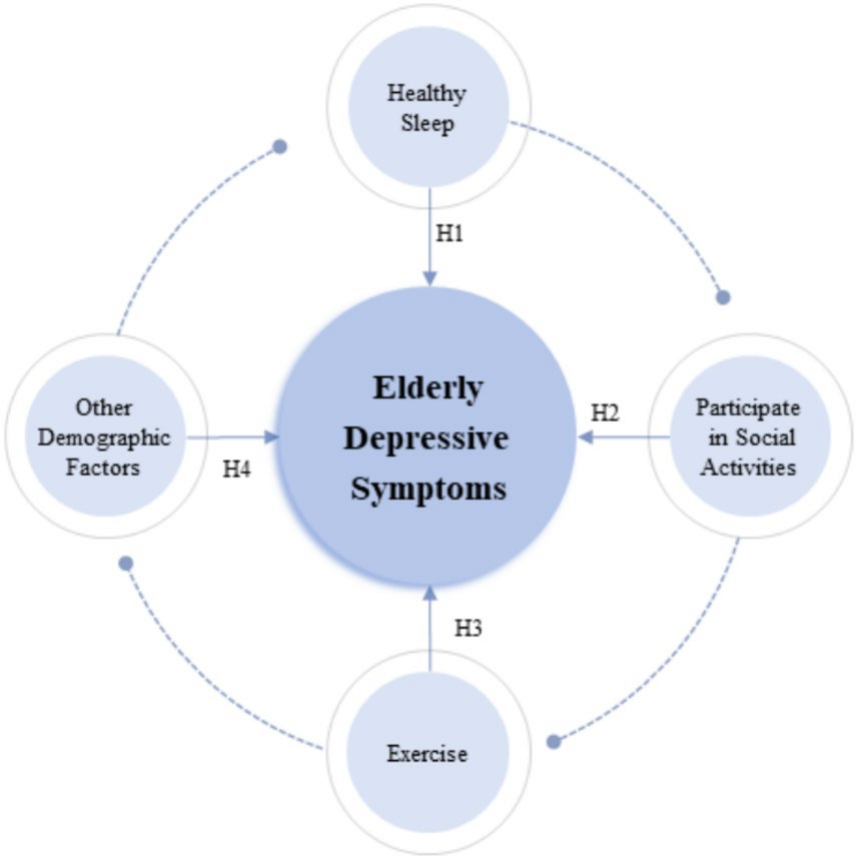
Main concepts and frame structure.

Building on a series of theoretical frameworks proposed by previous scholars, this study presents the following hypotheses (Figure 1) (30–37):

*H1:* The reasonableness of sleep duration (or whether sleep is healthy) is significantly correlated with the occurrence of depressive symptoms among the elderly.

Healthy sleep duration and habits can reduce the likelihood of depressive symptoms among the elderly; conversely, poor sleep habits may increase the risk of depressive symptoms in this population.

*H2:* Participation in social activities is significantly correlated with the occurrence of depressive symptoms among the elderly.

Participation in social activities can alleviate loneliness among the elderly, thereby promoting mental health and reducing the incidence of depressive symptoms.

*H3:* Engagement in physical exercise is significantly correlated with the occurrence of depressive symptoms among the elderly.

Regular physical exercise is considered a key component of a healthy lifestyle, which can significantly promote mental health and reduce the incidence of depressive symptoms among the elderly.

*H4:* Other demographic factors also influence the occurrence of depressive symptoms among the elderly.

## 3 Materials and methods

### 3.1 Data source

The data used in this study are drawn from the 2020 wave of the China Health and Retirement Longitudinal Study (CHARLS 2020). CHARLS is a nationally representative, large-scale household survey led by the National School of Development at Peking University, covering 28 provinces (including municipalities and autonomous regions) and 450 villages across China. The CHARLS questionnaire encompasses a wide range of personal and family information for middle-aged and elderly individuals, including depressive symptoms, cognitive function, self-rated health, and other variables representing physical and mental health, as well as demographic variables such as gender, age, marital status, and education level (16). These data provide substantial support for exploring the relationship between healthy lifestyle behaviors and depressive symptoms among the elderly in China. In this study, the data were cleaned according to the research objectives, excluding samples of individuals under the age of 60, as well as data that lacked information on healthy lifestyle behaviors, depressive symptoms, or relevant demographic characteristics. Ultimately, 9,020 valid samples were retained for analysis.

This study specifically focused on the elderly population aged 60 and above, as this group is more susceptible to depressive symptoms and is of particular interest in public health interventions. The exclusion of individuals under the age of 60 was based on the objective to clearly delineate the influence of healthy lifestyle behaviors on depressive symptoms in an aging population. While this exclusion criterion helps in tailoring the study’s findings to the targeted demographic, it may limit the generalizability of the results to younger populations. Future studies could explore similar relationships in younger cohorts to expand the applicability of the findings.

**Figure 2:**
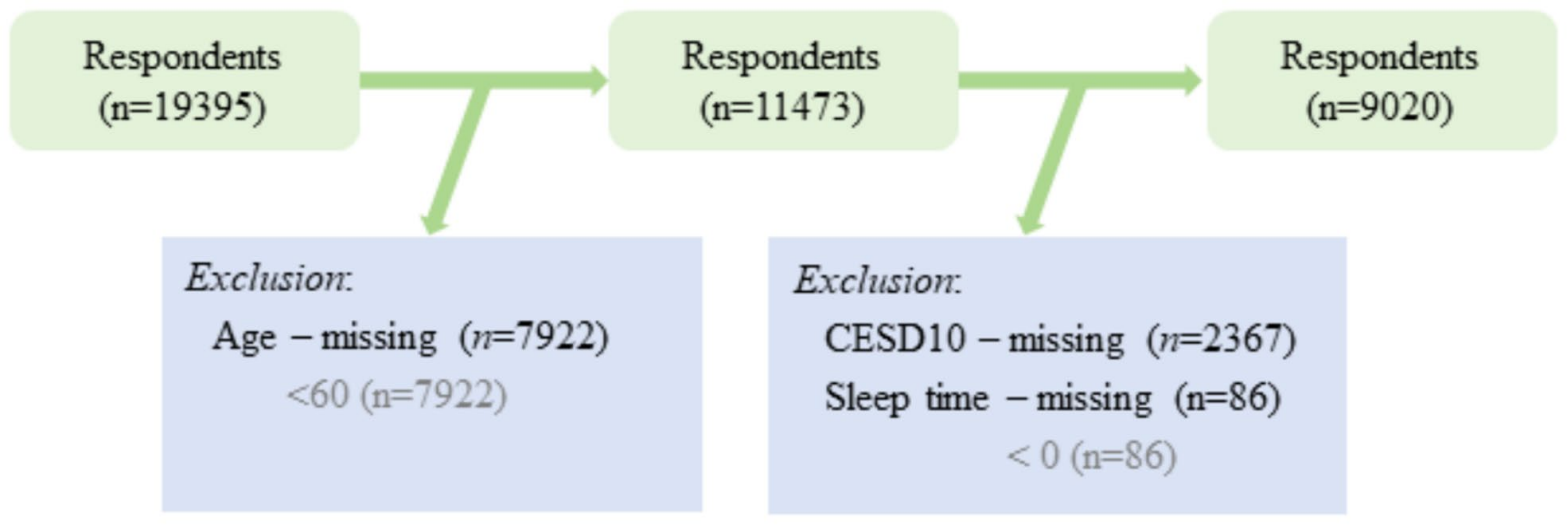
CHARLS 2020 sample flow chart.

Regarding the handling of missing data, the direct deletion method was employed primarily due to its simplicity and the minimal risk it poses to the integrity of the dataset given the large sample size. However, this method may introduce biases if the missing data are not completely random. An acknowledgment of this potential bias has been added, and the implications for the study’s findings are discussed. Future research could consider multiple imputation techniques to handle missing data, which may provide a more robust analysis by accounting for the randomness of missing data (see Figure 2).

### 3.2 Variable selection

#### 3.2.1 Life behavior style

According to Anderson (38), healthy lifestyle behaviors refer to actions taken by individuals to maintain and promote their health. In this study, we assess lifestyle health across three dimensions: “Whether sleep duration is adequate,” “Whether individuals participate in social activities,” and “Whether individuals engage in physical exercise.” Existing research suggests that moderate alcohol consumption may have positive effects on certain aspects of mental health, such as reducing stress and facilitating social interaction, while moderate smoking may temporarily alleviate stress or anxiety. Although CHARLS collected basic information on whether participants smoked or drank alcohol, it did not provide detailed data on excessive consumption. Due to the limitations of the dataset and other factors, smoking and drinking behaviors were excluded from the criteria used to assess whether a lifestyle was considered healthy (33, 39).

Based on the CHARLS questionnaire, the first indicator is the average natural sleep duration per night over the past month, as reported in the survey. Although the recommended average sleep duration for adults is more than 6 h per night, sleep patterns can vary widely among individuals. After excluding outliers, such as sleep durations less than 0 h, respondents with sleep durations between 6 and 10 h were classified as having “1: healthy sleep” while those with less than 6 h or more than 10 h were categorized as having “0: unhealthy sleep” forming the indicator for “whether sleep duration is reasonable.”

The second indicator is derived from eight questions in the questionnaire, including: (1) “whether participants visit or socialize with friends,” (2) “whether they play mahjong, chess, cards, or participate in community activities,” (3) “whether they provide unpaid help to relatives, friends, or neighbors who do not live with them,” (4) “whether they visit parks or other locations to engage in physical activities such as dancing or practicing Qigong,” and (5) “whether they participate in community activities.” etc. Participants who engaged in any of these activities were coded as “1” and those who did not participate in any were coded as “0” thus forming the indicator for “whether they participate in social activities.”

The third indicator includes three questions from the questionnaire: (1) “Do you engage in continuous strenuous exercise for at least 10 min per week?” (2) “Do you engage in moderate-intensity exercise for at least 10 min per week?” and (3) “Do you take a walk at least once a week?” Respondents who engaged in any of these activities were coded as “1” and those who did not were coded as “0” creating the indicator for “whether they engage in physical exercise” (40, 41).

#### 3.2.2 Depressive symptoms

The CHARLS questionnaire employs the CESD-10 scale to assess the risk of depression among the elderly. The scale consists of 10 items that reflect the feelings and behaviors of respondents over the past week, including: (1) “I am troubled by things that usually do not bother me,” (2) “It is difficult for me to concentrate on what I am doing,” (3) “I feel depressed,” (4) “I feel that everything I do requires great effort,” (5) “I am hopeful about the future,” (6) “I feel fearful,” (7) “I experience restless sleep,” (8) “I feel happy,” (9) “I feel lonely,” and (10) “I cannot ‘get going.’” Each item is rated on a four-point Likert scale: “rarely or none of the time” “some or a little of the time” “occasionally or a moderate amount of time” and “most or all of the time” corresponding to scores of 0, 1, 2, and 3, respectively. Total scores on the CESD-10 scale range from 0 to 30, with scores above 10 indicating the presence of depressive symptoms. In surveys of elderly populations in China, the scale has demonstrated high validity and reliability. To construct an ordered logistic regression model, this study categorized the scores as follows: 0–10 as “0: no depressive symptoms,” 11–20 as “1: mild depressive symptoms,” and 21–30 as “2: severe depressive symptoms” (42).

#### 3.2.3 Demographic variable

To more accurately assess and explain the significance of the influence of independent variables on dependent variables, this study incorporates demographic variables such as gender, age, education level, residence, and marital status. In the CHARLS questionnaire, education level is categorized into eight groups: illiteracy, incomplete primary school, self-study/tutoring, primary school, junior high school, senior high school, technical secondary school, and junior college or above. Marital status is classified into six categories: married with spouse present, married but temporarily not living with spouse due to work or other reasons, separated, divorced, widowed, and never married. Residence is divided into two categories: urban or rural (43, 44).

### 3.3 Statistical analysis method

In this study, Stata/MP 18.0 and SPSS 24.0 were used to clean and analyze the data. First, Stata/MP 18.0 was employed to clean the data according to the research objectives, excluding samples of individuals under the age of 60 and cases with missing values in relevant variables, resulting in a final dataset of 9,014 valid samples. Second, SPSS 24.0 was used to describe the remaining valid samples and to present the basic structure of the dataset. Third, chi-square tests and correlation analysis were conducted to examine the influence and relationships between demographic variables—such as lifestyle behaviors, education, marital status, and place of residence and the occurrence of depressive symptoms in the elderly. Finally, an ordered logistic regression model was constructed to analyze the relationship between healthy lifestyle behaviors and depressive symptoms among the elderly.

In this study, the ordered logistic regression model was adopted, because the symptoms of depression showed orderly grades (such as no depression, mild depression, and severe depression). Ordered logistic regression model is suitable for dealing with this kind of variables, which enables us to explore the influence of different lifestyle behaviors and socio-economic variables on depression levels. The coding of each variable is based on its data properties and practical significance. For example, variables such as gender, whether to exercise and whether to participate in social activities are all binary variables, so they are coded with 0 and 1, which is convenient for model calculation and interpretation. For multi-level variables such as income level, self-rated health status, life satisfaction, etc., orderly coding (such as 1 to 5 points) is adopted, so that the model can identify the differential effects of different levels of variables on depressive symptoms. In this study, the method of direct deletion is adopted for missing data. Deleting the missing data directly can keep the analysis simple and avoid the additional deviation that may be introduced by interpolation method. In addition, the sample size after deleting the missing data is still large enough to ensure the robustness of statistical analysis.

The specific formula for the ordered logistic regression model is as follows (Equations 1, 2):


(1) $$\begin{array}{l} \mathrm{logit}\left[\begin{array}{l} P\left(\mathrm{Depressive symptoms}<=0.0\right)/\\ \left(1-P\left(\mathrm{Depressive symptoms}<=0.0\right)\right) \end{array}\right]\\ =-0.734–0.896^{*}\mathrm{Healthy sleep}-0.107^{*}\mathrm{Participate}\;\\ \mathrm{in social activities}-0.150^{*}\mathrm{Exercise}\\ -0.523^{*}\mathrm{Gender}+0.576^{*}\mathrm{Residence}-0.338^{*}\mathrm{Marital status}\\ -0.231^{*}\mathrm{Degree of education}-0.041^{*}\mathrm{Age} \end{array}$$

(2) $$\begin{array}{l} \mathrm{logit}\left[\begin{array}{l} P\left(\mathrm{Depressive symptoms}<=1.0\right)/\\ \left(1-P\left(\mathrm{Depressive symptoms}<=1.0\right)\right) \end{array}\right]=1.443\\ –0.896^{*}\mathrm{Healthy sleep}-0.107^{*}\mathrm{Participate in social}\;\\ \mathrm{activities}-0.150^{*}\mathrm{Exercise}-0.523^{*}\mathrm{Gender}+0.576\\ {*}^{\mathrm{Residence}}-0.338^{*}\mathrm{Marital status}-0.231^{*}\mathrm{Degree of}\;\\ \mathrm{education}-0.041^{*}\mathrm{Age} \end{array}$$

The ordered logistic regression model was selected to analyze the relationship between healthy lifestyle behaviors and depressive symptoms, as the dependent variable, depressive symptoms score, is ordinal. This model is particularly suitable for data where the dependent variable is categorized ordinally, leveraging the natural order of the categories. It is preferred over linear regression when handling categorical outcomes and nonlinear relationships, especially when the dependent variable is not continuous. Moreover, it accounts for threshold probabilities between categories, allowing for a more accurate estimation of the transition probabilities between categories. The fundamental assumption of the model, the proportional odds assumption, posits that the explanatory variables exert a consistent effect across all binary splits of the outcome categories.

## 4 Analysis and discussion

### 4.1 Sample description

This study is a cross-sectional analysis focusing on individuals aged 60 and above from the CHARLS 2020 survey. After eliminating missing values and performing a series of data cleaning processes using Stata/MP 18.0, a total of 9,020 valid samples were obtained. Table 1 presents the basic structure of the valid samples.

**Table 1:** Distribution of valid survey samples.

Variable		Number / cases	Percentage
Gender	Male	4,539	50.32
	Female	4,481	49.68
Age	60–69 years old (inclusive)	5,361	59.43
	70–79 years old (inclusive)	3,027	33.56
	80–89 years old (inclusive)	608	6.74
	90 years old and above	24	0.27
Degree of education	Below primary school	4,303	47.71
	Primary school	1944	21.55
	Middle school	1,650	18.29
	Above Middle school	1,123	12.45
Marital status	Not in marriage	1739	19.28
	Married	7,281	80.72
Residence	Rural	5,352	59.33
	Urban	3,668	40.67
Exercise	Yes	8,061	89.37
	No	959	10.63
Healthy sleep	Yes	5,380	59.65
	No	3,640	40.35
Participate in social activities	Yes	4,276	47.41
	No	4,744	52.59
Depressive symptoms	No	5,786	64.15
	Mild	2,607	28.90
	Severe	627	6.95

According to Table 1, the sample consists of 4,539 males, accounting for 50.32% of the total, and 4,481 females, accounting for 49.68%. The gender distribution is nearly balanced, with a slightly higher proportion of males. Most of the sample is concentrated in the 60–69 age group, representing 59.43%, followed by the 70–79 age group at 33.56%. There are 608 individuals aged 80–89 (6.74%) and only 24 individuals aged 90 and above (0.27%), indicating that the sample primarily comprises elderly individuals between 60 and 79 years old.

In terms of educational level, 47.71% of the sample has an education level below primary school, 21.55% completed primary school, 18.29% have a middle school education, and 12.45% have a high school education or above. The data suggest that most elderly individuals have a low level of education.

Regarding marital status, 80.72% of the sample is married, while 19.28% is either unmarried or not in a marital relationship, indicating that most of the sample has partner support. Additionally, 59.33% of respondents reside in rural areas, while 40.67% live in urban areas, with a slightly higher proportion in rural areas, reflecting that the data includes more elderly individuals in rural areas.

In terms of exercise habits, 89.37% of respondents engage in regular exercise, while 10.63% do not. This suggests that most of the sample maintains an exercise habit, which may positively impact their physical health. Regarding sleep, 59.65% of respondents report having healthy sleep, while 40.35% report unhealthy sleep, indicating that nearly 40% of elderly individuals face sleep issues. Social activities are participated in by 47.41% of respondents, while 52.59% do not engage in social interactions, suggesting that more than half of the elderly population lacks social interaction, which may affect their mental health.

In terms of depressive symptoms, 64.15% of respondents show no depressive symptoms, 28.90% exhibit mild depressive symptoms, and 6.95% exhibit severe depressive symptoms, indicating that a certain proportion of elderly individuals experience depression. Regarding income, 32.92% of the sample is in the “very low” income level, 12.72% in the “low” level, 8.25% in the “middle-low” level, 11.27% in the “middle-high” level, 19.95% in the “high” level, and 14.89% in the “very high” level, with a higher proportion of the sample falling within the low to middle income levels.

### 4.2 Chi-square test

The chi-square test is a nonparametric statistical method used to analyze categorical data. It is primarily employed to examine the correlation or association between categorical variables. Its functions include testing whether two categorical variables are independent of each other, such as determining whether there is a significant association between gender and purchasing behavior. Additionally, it can be used for goodness-of-fit tests to assess whether the observed frequency distribution significantly differs from the theoretically expected distribution. By calculating the chi-square value, we can evaluate whether the observed differences are statistically significant, thereby determining whether to reject the null hypothesis and make inferences based on the study findings (45).

In this study, the chi-square test was used to examine the association (or independence) between depressive symptoms and eight variables: gender, age, education level, marital status, residence, exercise, participation in social activities, and healthy sleep. As shown in Table 2, the results indicate that the differences in depressive symptoms were statistically significant for all eight variables (*p* < 0.05).

**Table 2:** Chi square test results.

Variables		Depressive symptoms			*χ* ^2^	*p*
		No	Minor	Major		
Gender	Female	2,507 (43.33%)	1,540 (59.07%)	434 (69.22%)	281.095	0.000^***^
	Male	3,279 (56.67%)	1,067 (40.93%)	193 (30.78%)		
Age	60–69	3,522 (60.87%)	1,477 (56.65%)	362 (57.74%)	23.801	0.001^***^
	70–79	1853 (32.03%)	940 (36.06%)	234 (37.32%)		
	80–89	392 (6.77%)	185 (7.10%)	31 (4.94%)		
	90 and above	19 (0.33%)	5 (0.19%)	0 (0.00%)		
Degree of education	Below primary school	2,398 (41.44%)	1,486 (57.00%)	419 (66.83%)	353.252	0.000^***^
	Primary school	1,271 (21.97%)	555 (21.29%)	118 (18.82%)		
	Middle school	1,205 (20.83%)	380 (14.58%)	65 (10.37%)		
	Above middle school	912 (15.76%)	186 (7.13%)	25 (3.99%)		
Marital status	Not in marriage	951 (16.44%)	579 (22.21%)	209 (33.33%)	124.013	0.000^***^
	Married	4,835 (83.56%)	2028 (77.79%)	418 (66.67%)		
Residence	Urban	2,676 (46.25%)	820 (31.45%)	172 (27.43%)	211.963	0.000^***^
	Rural	3,110 (53.75%)	1787 (68.55%)	455 (72.57%)		
Exercise	No	551 (9.52%)	300 (11.51%)	108 (17.22%)	38.276	0.000^***^
	Yes	5,235 (90.48%)	2,307 (88.49%)	519 (82.78%)		
Healthy sleep	No	1870 (32.32%)	1,332 (51.09%)	438 (69.86%)	506.828	0.000^***^
	Yes	3,916 (67.68%)	1,275 (48.91%)	189 (30.14%)		
Participate in social activities	No	2,947 (50.93%)	1,429 (54.81%)	368 (58.69%)	20.905	0.000^***^
	Yes	2,839 (49.07%)	1,178 (45.19%)	259 (41.31%)		

^***^ represents the significance level of 1%.

### 4.3 Sensitivity analysis

Because “whether sleep duration is adequate” and CESD’s sleep item (7) “I experience restless sleep” are related to sleep quality, there is a possibility of overlap, which may lead to the obvious relationship between depression symptoms and sleep time being artificially exaggerated. Therefore, it is necessary to carry out sensitivity analysis to analyze the independent effects of “whether sleep duration is adequate” and “restless sleep” on the depressive symptoms of the elderly. This analysis can help us distinguish the independent effects of the two on depressive symptoms more clearly, and avoid the confusion caused by the overlapping of dependent variables, thus making the research results more stable and reliable. The sensitivity analysis results are shown in Table 3.

**Table 3:** Sensitivity analysis results.

	Depressive symptoms			
	Coefficient of regression	95% CI	VIF	Tolerance
CESD(7)	−0.481***(−9.691)	−0.579 ~ −0.384	2.142	0.467
Healthy sleep	−2.259***(−11.517)	−2.644 ~ −1.875	2.142	0.467
Cons	13.343***(56.769)	12.882 ~ 13.804	–	–
*N*	9,020			
*R* ^2^	0.085			
Adjust *R*^2^	0.084			
*F* value	*F* (2, 9,017) = 417.095, *p* = 0.000***			
D-W Value	1.686			

^***^ represents the significance level of 1%. Values in brackets are *t* value.

In the sensitivity analysis, we made regression analysis on “whether the sleep time is enough” and “restless sleep” respectively, and found that both of them were significantly related to the depressive symptoms of the elderly. This result shows that sleep time and sleep quality have independent predictive effects on depressive symptoms, and their effects on depressive symptoms are not completely overlapping. This further shows that even if the subjective sleep quality is excluded, adequate sleep time itself still has a significant protective effect on the depressive symptoms of the elderly. Similarly, as an evaluation index of subjective sleep quality, sleep anxiety can independently predict the changes of depressive symptoms. This finding supports the value of sleep time and sleep quality as independent variables in the study of depressive symptoms, and provides a clearer measurement direction for future research.

### 4.4 Correlation analysis

Pearson correlation analysis is a statistical method used to measure the strength and direction of the linear relationship between two continuous variables. This method helps to determine the degree of correlation between variables. By calculating the correlation coefficient, we can assess whether the relationship between variables is positive, negative, or nonexistent.

In addition, Pearson correlation analysis can evaluate the strength of the correlation, making it widely used across various research fields, particularly when exploring the presence of a significant linear relationship between different factors. This method is commonly applied in disciplines such as economics, psychology, and the social sciences. By determining whether the relationship between variables is statistically significant, Pearson correlation analysis provides a foundation for further analysis (46).

In this study, correlation analysis was used to examine the relationships between eight variables: gender, age, education level, marital status, residence, exercise, participation in social activities, and healthy sleep, etc. Pearson correlation coefficients were calculated to express the strength of these relationships. The specific analysis is presented in Table 4.

**Table 4:** Pearson correlation analysis results.

		Depressive symptoms
Gender	Correlation coefficient	−0.175
	*p* value	0.000^***^
Age	Correlation coefficient	0.020
	*p* value	0.056
Degree of education	Correlation coefficient	−0.195
	*p* value	0.000^***^
Marital status	Correlation coefficient	−0.115
	*p* value	0.000^***^
Residence	Correlation coefficient	0.148
	*p* value	0.000^***^
Exercise	Correlation coefficient	−0.061
	*p* value	0.000^***^
Healthy sleep	Correlation coefficient	−0.237
	*p* value	0.000^***^
Participate in social activities	Correlation coefficient	−0.048
	*p* value	0.000^***^

^***^ represents the significance level of 1%.

### 4.5 Ordered logistic regression analysis

Ordered logistic regression analysis is a regression method used to handle data with ordered dependent variables. Its primary function is to help researchers analyze and predict the relationships between different categories of dependent variables and to explore the influence of independent variables on ordered dependent variables (47).

In this study, we first analyze the overall effectiveness of the model using the model likelihood ratio test. As shown in Table 5, the null hypothesis of the test is that the inclusion or exclusion of independent variables (Healthy Sleep, Participation in Social Activities, Exercise, Gender, Residence, Marital Status, Education Level, and Age) does not affect the quality of the model. The analysis results indicate that the null hypothesis is rejected (*χ*^2^ = 1092.705, *p* = 0.000 < 0.05), meaning that the independent variables included in the model are effective, and the model construction is meaningful.

**Table 5:** Likelihood ratio test of ordered logistic regression model.

Model	−2*x* log-likelihood value	Chi square value	df	*p*	AIC value	BIC value
Intercept only	14953.333	–	–	–	–	–
Final model	13860.656	1092.677	8	0.000^***^	13880.656	13951.728

^***^ represents the significance level of 1%.

Next, we used the aforementioned eight factors as independent variables and depressive symptoms as the dependent variable for ordered logistic regression analysis. The pseudo-R-squared value (McFadden R-squared) of the model is 0.073, indicating that these eight factors explain 7.3% of the variation in depressive symptoms. The summary of analysis results of ordered Logistic regression model are shown in Table 6.

**Table 6:** Summary of analysis results of ordered logistic regression model.

Variables		Coefficient of regression	Standard error	*z* value	Wald *χ*^2^	*p* value	OR value	OR value 95% CI
Threshold of dependent variable	No	−0.734	0.103	−7.096	50.359	0.000^***^	2.084	1.702 ~ 2.553
	Mild	1.443	0.107	13.519	181.775	0.000^***^	0.236	0.192 ~ 0.291
Independent variable	Healthy sleep	−0.896	0.046	−19.606	384.411	0.000^***^	0.408	0.373 ~ 0.447
	Participate in social activities	−0.107	0.046	−2.324	5.400	0.020^**^	0.899	0.821 ~ 0.983
	Exercise	−0.150	0.071	−2.102	4.418	0.036^**^	0.861	0.749 ~ 0.990
	Gender	−0.523	0.048	−10.813	116.921	0.000^***^	0.593	0.539 ~ 0.652
	Residence	0.576	0.049	11.654	135.823	0.000^***^	1.780	1.615 ~ 1.961
	Marital status	−0.338	0.057	−5.886	34.640	0.000^***^	0.713	0.637 ~ 0.798
	Degree of education	−0.231	0.024	−9.459	89.470	0.000^***^	0.794	0.757 ~ 0.833
	Age	−0.041	0.037	−1.105	1.220	0.269	0.960	0.893 ~ 1.032

^***^ represents the significance level of 1%, ^**^ represents the significance level of 5%.

In this study, the model’s fitting quality is assessed by its prediction accuracy. As shown in Table 7, the overall prediction accuracy of the model is 64.98%, indicating that the model’s fit is acceptable.

**Table 7:** Prediction accuracy of ordered logistic regression model.

Depressive symptoms variables	Actual frequency	Prediction accuracy frequency	Prediction accuracy (%)
No	5,786	5,316	91.877
Minor	2,607	545	20.905
Severe	627	0	0.000
Total	9,020	5,861	64.978

The empirical results of this study demonstrate that maintaining healthy sleep is significantly associated with the incidence of depressive symptoms in the elderly. Similarly, active participation in social activities and maintaining regular exercise habits also significantly associated with the likelihood of depressive symptoms in this population. These findings validate the three hypotheses proposed in this paper, indicating that healthy lifestyle behaviors is associated with lower the risk of depressive symptoms among the elderly. Based on the empirical analysis, it can be inferred that healthy lifestyle behaviors contribute to maintaining and improving brain function. Exercise, in particular, has been shown to increase endorphin levels, which enhances mood and promotes a sense of well-being. Additionally, adopting a healthy lifestyle provides positive psychological reinforcement, leading to increased self-efficacy and self-worth, thereby reducing the likelihood of anxiety and depression. Active participation in social activities also provides emotional support, alleviating feelings of loneliness and social isolation, which are known risk factors for depressive symptoms. By improving physical health and self-care abilities, elderly individuals experience greater life satisfaction and reduced negative emotions, which, in turn, further lowers the incidence of depressive symptoms (48–51).

In the analysis of the relationship between healthy lifestyle behaviors and depressive symptoms, the odds ratios (ORs) and their 95% confidence intervals (CIs) were calculated to quantify the strength and direction of these associations. The results indicated that adequate sleep was associated with a 59.2% reduction in the odds of depressive symptoms (OR = 0.408, 95% CI: 0.373–0.447). Regular physical exercise was linked to a 13.9% decrease in the odds of depressive symptoms (OR = 0.861, 95% CI: 0.749–0.990). Additionally, active social participation showed a 10.1% lower odds of depressive symptoms (OR = 0.899, 95% CI: 0.821–0.983). These findings suggest that engaging in healthy lifestyle behaviors can significantly mitigate the risk of depressive symptoms among the elderly.

The sensitivity analysis conducted on sleep duration and quality highlighted the nuanced impact of these factors on depressive symptoms. The results demonstrate that not only the quantity but also the quality of sleep significantly influences mental health among the elderly. Specifically, the analysis showed that both insufficient and excessive sleep durations are linked to an increased risk of depressive symptoms, while optimal sleep duration (as identified in the study) correlates with a reduced risk. Furthermore, the quality of sleep, as reflected in the measures of sleep disturbances included in the CESD-10 scale, independently contributes to the likelihood of depressive symptoms, regardless of sleep duration. This finding underscores the importance of considering both aspects of sleep in interventions aimed at reducing depressive symptoms. Integrating these results into the overall conclusions, it becomes evident that promoting both adequate sleep duration and quality are essential for mental health interventions. This study thus supports the growing body of literature suggesting that comprehensive sleep management should be a key component of public health strategies aimed at the elderly population, emphasizing not only the duration but also the quality of sleep.

Demographic characteristics, such as place of residence and gender, were also included in this study. First, the empirical results confirm the correlations of these demographic characteristics on the occurrence of depressive symptoms among the elderly. The results show that living in rural areas or cities is significantly related to the occurrence of depression in the elderly. This may be attributed to the fact that urban residents have better access to medical resources, a wider range of social activities, and more diverse cultural and recreational facilities, all of which contribute to enhanced life satisfaction and a reduced likelihood of depression. Second, the study found that older women are more likely to experience depressive symptoms compared to men, possibly due to physiological differences. In particular, hormonal changes, such as decreased estrogen levels during and after menopause, may impair emotional regulation, thereby increasing the risk of depressive symptoms. Third, the analysis revealed that married individuals have a lower risk of developing depressive symptoms than their unmarried counterparts. This may be because marriage provides stable emotional support and companionship. The presence of a spouse offers psychological support in daily life, mitigating feelings of loneliness and social isolation. Finally, the study also showed that individuals with higher levels of education are less likely to experience depressive symptoms. This could be because those with higher education tend to have better psychological resilience and more effective coping strategies, enabling them to manage life’s challenges and setbacks more constructively (52–55).

The analysis of urban–rural differences in depressive symptoms revealed significant disparities, which this study has initially addressed. However, the broader implications of socio-economic status and healthcare resource availability between urban and rural areas warrant further discussion. Urban areas typically have better healthcare infrastructure, more accessible mental health services, and greater awareness of mental health issues, which can contribute to lower prevalence rates of depressive symptoms compared to rural areas. Conversely, rural areas often face challenges such as limited access to healthcare services, lower health literacy, and less exposure to health promotion activities, which can exacerbate mental health issues. Furthermore, socio-economic factors such as income levels, employment status, and educational attainment also play crucial roles in mental health disparities. Higher socio-economic status often correlates with better access to healthcare resources and preventive services, which can mitigate risks associated with depressive symptoms. This study suggests that public health interventions aimed at reducing depressive symptoms should consider these disparities, tailoring strategies to address specific needs in urban and rural settings. For example, enhancing healthcare access and mental health awareness in rural areas could be a priority, while urban strategies might focus on managing the stresses associated with urban living.

Many studies support that a healthy lifestyle can help reduce the risk of depression. For example, a large prospective cohort study found that positive lifestyle habits, such as adequate sleep and high level of physical activity, were significantly associated with lower risk of depression recurrence and death (56). In addition, healthy diet, diversified nutrition intake and regular social interaction are helpful to enhance mental health and reduce the occurrence of depression (57). These results are consistent with the conclusion of this study, which further verifies the key role of healthy lifestyle in depression prevention.

However, the results of this study are different from some literatures in terms of urban–rural differences and the role of social activities. For example, this study shows that the elderly in rural areas are more susceptible to depressive symptoms, which may be caused by insufficient medical resources and weak social support system in rural areas. On the contrary, some studies have not found significant differences between urban and rural areas, especially in countries with small differences in economic situation and cultural background. This may be because some rural communities have closer social networks and higher community participation, which reduces the risk of loneliness and depression. Similarly, in terms of the influence of social participation, the results of this study show that it has a significant impact on depressive symptoms, but some literatures have not reached the same conclusion. This difference may be due to the different definitions of social participation: some studies only look at formal social activities, while this study includes family support and informal social interaction (58–60).

The analysis of urban–rural disparities in depressive symptoms within this study highlights significant socio-economic and healthcare access issues that align with global findings. Research from Africa points to the crucial need for multi-faceted interventions to bridge the gap in mental health care between urban and rural areas, emphasizing the importance of government intervention and community involvement to develop sustainable mental health care infrastructure (61). These findings resonate with studies from the United States, which have documented how social determinants of health contribute significantly to the disparities in mental health outcomes between rural and urban populations (62). Additionally, policy recommendations stress the importance of expanding mental health services in rural areas to include a broader network of providers and to address the stigma associated with seeking mental health care (63). By integrating these insights, this study not only echoes the challenges faced globally in addressing urban–rural mental health disparities but also underlines the necessity of targeted public health strategies that cater to the specific needs of rural populations. These discussions suggest that, like in other parts of the world, China’s rural areas might benefit significantly from policies that focus on enhancing healthcare infrastructure and reducing the socioeconomic disparities that exacerbate mental health issues. This comparative analysis offers a broader understanding of the universal challenges and strategic interventions needed to mitigate these disparities effectively.

The authors believes that there are many potential reasons for the differences, which may include the following three factors: cultural and social background, multi-dimensional influence of healthy lifestyle and different research methods. In terms of cultural and social background, China’s cultural background emphasizes family support, and the popularization of empty nest phenomenon may aggravate the loneliness and psychological pressure of the elderly. In contrast, in western society, the elderly are usually more independent and less dependent on family support, which leads to different functions of cultural background in depressive symptoms. In the multi-dimensional impact of healthy lifestyle, the authors thinks that different studies have different definitions of healthy lifestyle. For example, some studies have included multi-dimensional health factors such as drinking, smoking and diet quality, while this study only focuses on sleep, social activities and physical exercise. In terms of research methods, some studies adopt longitudinal design, which is more suitable for discussing causality, but the cross-sectional data used in this study may not fully explain the complexity of causality. In addition, the difference of sample characteristics and the time of data collection may also lead to inconsistent results. Based on the above viewpoints, the authors believes that the reasons for the differences can be reasonably explained.

The findings of this study supplement and expand upon existing research. The results suggest that encouraging the elderly to actively participate in social activities, maintain regular exercise habits, and adopt healthy sleep routines can help reduce the incidence of depressive symptoms (64).

## 5 Conclusion

Using data from the 2020 China Health and Retirement Longitudinal Study (CHARLS 2020), this study explored the relationship between healthy lifestyle behaviors and the incidence of depressive symptoms among the elderly. The results indicate that maintaining healthy sleep, actively participating in social activities, and regularly engaging in physical exercise play a significant role in reducing depressive symptoms among the elderly in China.

These findings are of great significance for public health policy aimed at improving the mental health of the elderly population in China. By promoting healthy lifestyles, particularly those related to healthy sleep, social interaction, and physical exercise, it is possible to reduce the mental health burden faced by the elderly and lower the risk of depressive symptoms. In addition, the study also considered the impact of demographic factors, such as marital status and education level, which further confirmed their influence on depressive symptoms among the elderly.

In our study, a large number of participants (*n* = 7,922) were excluded due to lack of age information or under 60 years old. This exclusion is necessary, because our research is specifically aimed at the elderly population, and the elderly are defined as people aged 60 and above. This introduces a potential selection bias. This deviation may limit the applicability of our research results to a wider elderly population. Future research can improve this point by ensuring more comprehensive demographic data collection, so as to analyze the influence of lifestyle behavior on different elderly population groups more widely. We admit that this is a limitation. Although this limitation limits the universality of our results, our research results are still helpful to understand how lifestyle factors affect the depression of the elderly, especially those who meet our research age standards.

Although this research has made valuable contributions to public health and psychology, it still has several limitations. For instance, the study adopts a cross-sectional design, which limits the ability to infer causality. Additionally, variables such as diet and economic income were not considered, and longitudinal data are lacking. Future research could explore these additional factors and apply a longitudinal design to better understand the causal relationship between lifestyle behaviors and depressive symptoms, potentially using survey data from other years in CHARLS.

The findings of this study not only provide empirical support for understanding the relationship between healthy lifestyle and depressive symptoms of the elderly, but also provide valuable reference for the formulation of public health intervention measures.

First of all, the research results show that the incidence of depressive symptoms can be effectively reduced by promoting a healthy lifestyle, such as encouraging the elderly to maintain proper sleep habits, participate in social activities and exercise regularly. This provides a clear direction for policy makers, that is, emphasizing the promotion of healthy lifestyles in public health policies will help reduce the mental health burden of the elderly.

Secondly, community and medical services can also be adjusted based on the results of this study. For example, the community can promote social participation and improve life satisfaction by organizing various activities suitable for the elderly, thus alleviating the symptoms of depression. In addition, personalized intervention measures are the future development direction, and special health intervention plans can be made for high-risk groups, such as sleep management and social participation plans, in order to achieve targeted mental health improvement.

Finally, family support plays an important role in the mental health of the elderly. The research results can provide guidance for family nursing and help family members understand how to reduce the psychological burden of the elderly by encouraging healthy living habits, such as regular work and rest and participating in outdoor activities. Through these multi-level interventions, the results of this study are expected to be transformed into concrete practical applications, thus improving the mental health of the elderly in practice.

Overall, cultivating healthy lifestyle behaviors can significantly improve the mental health of the elderly. Policymakers, healthcare providers, community organizations, and families of the elderly should encourage older adults to adopt healthier living habits, contributing to the broader goal of promoting healthy aging in China.

## Data Availability

The datasets presented in this study can be found in online repositories. The names of the repository/repositories and accession number(s) can be found in the article/supplementary material.
